# Cognitive control depletion reduces pre-stimulus and recollection-related measures of strategic retrieval

**DOI:** 10.12688/wellcomeopenres.15347.2

**Published:** 2019-10-18

**Authors:** Jane E Herron

**Affiliations:** 1 Cardiff University Brain Research Imaging Centre (CUBRIC), School of Psychology, Cardiff University, Cardiff, CF24 4HQ, UK

**Keywords:** Memory, ERP, retrieval orientation, strategic retrieval, pre-retrieval, cognitive control

## Abstract

**Background: **The ability to strategically retrieve task-relevant information from episodic memory is thought to rely on goal-directed executive processes, and there is evidence that neural correlates of strategic retrieval are sensitive to reserves of cognitive control. The present study extended this work, exploring the role of cognitive control in the flexible orienting of strategic retrieval processes across alternating retrieval goals.

**Method: **Pre-stimulus cues directed participants to endorse memory targets from one of two encoding contexts, with the target encoding context alternating every two trials. Items from the nontarget encoding context were rejected alongside new items. One group of participants completed a Stroop task prior to the memory test in order to deplete their reserves of cognitive control, while a second group performed a control task. Event-related potentials (ERPs) were recorded throughout the memory task, and time-locked to both pre-stimulus cues and memory probes.

**Results:** Control participants’ pre-stimulus ERPs showed sustained divergences at frontal electrode sites according to retrieval goal. This effect was evident on the first trial of each memory task, and linked with the initiation of goal-specific retrieval orientations. Control participants also showed enhanced ERP correlates of recollection (the ‘left parietal effect’) for correctly classified targets relative to nontargets on the second trial of each memory task, indexing strategic retrieval of task-relevant information. Both the pre-stimulus index of retrieval orientation and the target/nontarget left parietal effect were significantly attenuated in participants that completed the Stroop task.

**Conclusions:** The reduction of pre-stimulus and stimulus-locked ERP effects following the Stroop task indicates that available reserves of cognitive control play an important role in both proactive and recollection-related aspects of strategic retrieval.

## Introduction

The retrieval of episodic information in accordance with current goals is enabled by an ensemble of memory control processes which occur prior to, during and after the reactivation of the episodic trace. Many of these processes are closely tied to ‘retrieval orientations’, goal-directed memory states that facilitate the retrieval of relevant contextual details ^1^. The real-time nature of event-related potentials (ERPs) allows memory control processes to be isolated according to their latency and functional properties. Task-switching memory studies have revealed that pre-stimulus cues signalling the onset of different source memory tasks elicit sustained slow-wave ERPs that diverge at frontal electrode sites according to retrieval goals ^2^. This effect occurs only when a new memory task begins, linking it with processes involved in the initiation of orientations (e.g. task-set configuration) rather than their maintenance throughout tasks ^3^. Importantly, these pre-retrieval measures predict whether criterial source judgments associated with the upcoming test items will be correct or incorrect ^4, 5^, indicating that pre-retrieval control processes could act as gateways to memory.

It is assumed that a retrieval orientation will be tonically maintained while that retrieval goal remains in place ^1^. Many electrophysiological and neuroimaging studies have tested this theory by contrasting neural activity elicited by new items between tasks with different retrieval goals, the assumption being that orientations influence how memory probes are processed. This approach allows task-specific processing of memory probes to be identified without confounding this with differences in retrieved content. A large body of ERP and fMRI research supports this view ^6– 24^, and an individual differences analysis demonstrated that this index is positively correlated with memory accuracy ^25^. However, this is an indirect measure of retrieval orientation, reflecting task-specific processes operating downstream from those directly involved in sustaining the orientation. This was confirmed by an fMRI study separating item-related neural activity from sustained supra-item neural activity, which found reliable and dissociable effects of retrieval goal in both measures ^26^.

More recently, it was reported that pre-stimulus ERPs elicited by a neutral fixation asterisk differed according to distinct retrieval goals which were each maintained throughout separate tasks, and it was proposed that this effect directly reflected the ongoing maintenance of retrieval orientations ^27^. One group of participants in this study completed a Stroop task ^28^ between study and test. This consists of colour names printed in incongruently coloured ink, and participants must name the colour of the ink. Overcoming this interference requires participants to exercise cognitive control, the depletion of which impairs performance on further tasks requiring cognitive control ^29, 30^ including the recollection of autobiographical memories ^31, 32^. The Stroop task eliminated the pre-stimulus ERP orientation effect observed in the control group, indicating that orientation maintenance depends on available reserves of cognitive control. Although memory accuracy was not impaired, the Stroop group showed enhanced ERP measures of post-stimulus monitoring at right frontal sites, indicating an increased reliance on post-retrieval processing in the absence of pre-retrieval orientations.

An earlier study by Elward *et al.* ^33^ examined how the Stroop task influenced ERP measures of strategic retrieval. Concrete nouns were encoded in two different tasks (specify the item’s function or rate how easy/difficult it would be to draw), and participants were instructed to accept test items from a specified encoding task on one key (‘targets’) while rejecting test items from the non-specified task (‘nontargets’) on the same key as unstudied items ^34^. ‘Strategic retrieval’ refers to the prioritised retrieval of goal-specific contextual information, and manifests as larger recollection effects for targets than for nontargets. The ERP correlate of recollection is enhanced positivity at parietal electrode sites between 500–800ms, and this is often significantly larger for targets than for nontargets ^12, 22, 35, 36^. Elward *et al.* ^33^ found that participants with high working memory capacity (measured using O-span performance) showed ERP evidence of strategic retrieval, but that this was eliminated following the Stroop task. In contrast, the more recent study described above observed ERP evidence of strategic retrieval that was equivalent across control and Stroop participants ^27^.

The present study examined the effects of cognitive control depletion on goal-directed memory retrieval further. The same encoding and retrieval tasks used in ERP studies described above ^27, 33^ were used here in conjunction with the Stroop manipulation. The two retrieval tasks were, however, interleaved in a task-switching memory paradigm, with pre-stimulus cues signifying the memory task. The purpose of this was twofold; a task-switching design would allow ERP measures of retrieval orientation *initiation* to be captured, and their susceptibility to cognitive control depletion evaluated. Second, switching between different memory tasks requires a greater degree of cognitive control, and it was predicted that ERP measures of strategic retrieval would consequently be more vulnerable to the Stroop manipulation. There were therefore two principal experimental hypotheses: i) participants completing the Stroop task would show attenuated pre-stimulus ERP measures of retrieval orientation initiation on the first trial of each memory task when compared with controls, and ii) item-locked ERP measures of strategic retrieval (i.e. greater positivity for targets than for nontargets between 500–800ms) would be evident in the control group but reduced in the Stroop group.

## Methods

### Participants

Participants were Cardiff University undergraduate students who responded to a request for experimental volunteers hosted by the institution’s online experimental management system. Inclusion criteria required participants to be aged 18–30, right-handed, speak native English, and have normal or corrected-to-normal vision. Participants gave informed written consent prior to the experiment and were remunerated for their time at a rate of £7.50/hr. Ethical approval was granted by Cardiff University’s School of Psychology ethics committee (approval number EC.15.08.11.4184GA2). The experimental dataset consisted of behavioural and electrophysiological data collected from 50 participants, and the data collection methods are detailed below. Two datasets were discarded due to excessive ERP artifacts, and the remaining 48 participants were alternately allocated to the Stroop group (N=24) or the control group (N=24). This sample size was determined by power calculations based on prior studies which indicated that a sample size of 24 will have 95% power to detect a retrieval orientation initiation ERP effect at p<0.05 at the group level (ω _p_²=0.10 ^3^), a sample size of 20 will have 95% power to detect a strategic retrieval ERP effect at p<0.05 (ωp²=0.14 ^27^), and that a sample size of 20 will have 95% power to detect a Stroop/control group x retrieval goal interaction in pre-retrieval ERPs at p<0.05 (η² _p_ = 0.016 ^27^). The control group (21 female) were aged 18–24 (mean age: 19.8 years), and the Stroop group (19 female) aged 18–30 (mean age: 21 years; t(1,23) = 1.31, p = 0.20, n.s.).

### Design

Data collection took place in CUBRIC’s EEG Lab2 between the 26 ^th^ October 2016 and the 25 ^th^ May 2017. Stimuli from the Medical Research Council (MRC) psycholinguistic database ^37^ consisted of 240 concrete and imageable nouns (integer normative values of concreteness ^38^ and imageability ^39^ both ranged from 550–700 ^37^) with a frequency of 1–15 per million ^40^ and a letter range of 3–10. Words were shown in white letters on a black computer screen 1.2m from the participant and were presented at central fixation with maximum visual angles of 0.5° (vertical) and 2.2° (horizontal). A single study phase was followed by the Stroop/control task and then by the memory test. Six lists of 40 words were created. Four lists were used in the study phase (160 words). Cues (“FUNCTION” or “DRAW”) preceding each study word specified the encoding task, each of which was performed for two consecutive trials. Participants either verbally stated the function of the item, or verbally stated whether the item would be easy or difficult to draw. Cues were presented for 300ms, the screen was blanked for 2000ms, then the study word presented for 300ms. Participants verbally responded before pressing a response key. The next study trial started 1000ms after this keypress. After study, Stroop participants read aloud from five sheets of A4 paper containing five columns of equiprobable colour names (‘red’, ‘green’, ‘blue’ and ‘yellow’) for 6.5 minutes ^27, 33^. Word colour and colour meaning were incongruent for 75% of items. Participants were instructed to state the ink colour and to prioritise accuracy over speed. Control participants were given colour names printed in black ink and were instructed to read these aloud.

The memory test contained all six wordlists (240 words); 80 words had been encoded in the function task, 80 in the drawing task, and 80 were new items. These were randomised and preceded either by a “FUNCTION?” or “DRAW?” cue. Each cue-type preceded equal numbers of each stimulus type and was presented for two consecutive trials. The memory test was a cued exclusion task; participants responded on one key to words studied in the specified encoding task (‘targets’) and on another key to both words from the non-specified encoding task (‘nontargets’) and new words. The hand used for each response type was counterbalanced across participants. Wordlists were counterbalanced so that each word served as a target, a nontarget and a new item following each cue-type an equal number of times. Test trials began with the ‘FUNCTION?’ or ‘DRAW?’ cue (300ms) followed by a blank screen (2000ms) and then the test word (300ms). The screen was blanked until the participant responded, and the next trial began 1000ms later.

### Electroencephalogram (EEG) acquisition

EEG was recorded from 32 active electrodes embedded in a cap using a BioSemi ActiveTwo Mk1 electrode system, which incorporated a BioSemi ActiveTwo AD-box ADC-8. Electrode locations were distributed across the scalp and were based on the International 10–20 system ^41^. These included midline (Fz, Cz, Pz, Oz,) and left/right hemisphere locations (FP1/FP2, F7/F8, F5/F6, F3/F4, F1/F2, T7/T8, C5/C6, C3/C4, C1/C2, P7/P8, P5/P6, P3/P4, P1/P2, O1/O2). Six further single electrodes were placed on both mastoid processes, above and below the left eye (vertical electrooculogram; VEOG) and from both outer canthi (horizontal electrooculogram; HEOG). EEG (range: DC-419 Hz) was sampled at 2048 Hz and referenced to two linked electrodes positioned midway between POz and PO3/PO4. EEG data was pre-processed using EEGLAB 14 ^42^, a non-proprietary MATLAB toolbox that can also be run using Octave. The continuous EEG data were downsampled to 200 Hz, bandpass filtered (0.1–30 Hz) using EEGLAB’s new finite impulse response filter and re-referenced to linked mastoids. Eye movement correction was applied using the extended ‘runica’ infomax independent component analysis ^42, 43^. Components reflecting blinks and saccadic eye movements were identified by visual examination of component topographies, time courses, and spectra. These were corrected by back-projecting all but these components to the data space. The ERPLAB toolbox ^44^ was then used to create cue-locked and stimulus-locked event-related potentials. These functions are also available within EEGLAB. Cue-locked ERPs were 2000ms in length with an additional 200ms pre-cue baseline. Stimulus-locked ERPs were 1500ms in length with an additional 200ms pre-stimulus baseline. Trials containing EEG artifact were eliminated using the ERPLAB moving window peak-to-peak threshold algorithm (voltage threshold = 100, moving windows = 200, window step = 50) and artifact rejection was then visually verified. The proportion of cue-locked ERP trials rejected due to artifact was 0.06 in both groups. The proportions of item-locked ERP trials rejected due to artifact were 0.05 in the control group and 0.04 in the Stroop group.

### Statistical analysis

All first-stage behavioural and electrophysiological analyses employed mixed model ANOVAs using a non-proprietary analysis program called ALAS (version ALASBIG.exe, archived on the Open Science Framework, see data availability statement), which incorporates the Greenhouse-Geisser non-sphericity correction ^45^. All reported results include the epsilon-corrected degrees of freedom. The design of each ANOVA is specified below under the relevant behavioural and ERP subheadings. The mean amplitude ERP values subjected to these analyses were calculated using the ERPLAB toolbox ^44^.

## Results

### Behavioural analyses

Accuracy data (see Table 1) and reaction time data ^46^ (see Table 2) were each analysed with a mixed model ANOVA incorporating the within-subjects factors of Retrieval Task (function/draw), Switch/Stay (first/second trial within each retrieval task), and Item Type (target/nontarget/new) and the between-subjects factor of Group (Stroop/control).

**Table 1.** Mean response accuracy (%) to each item type separated by Retrieval Task, Switch/Stay, and Group (Stroop/control). 95% confidence intervals in parentheses.

	Control		Stroop	
	Switch	Stay	Switch	Stay
***Function***				
**Target**	85 (± 4.0)	81 (± 4.0)	81 (± 4.8)	81 (± 6.4)
**Nontarget**	75 (± 6.4)	85 (± 4.4)	84 (± 4.4)	84 (± 4.8)
**New**	96 (± 1.6)	96 (± 2.0)	97 (± 1.6)	96 (± 2.8)
***Drawing***				
**Target**	78 (± 6.4)	80 (± 4.4)	83 (± 4.4)	83 (± 4.0)
**Nontarget**	78 (± 4.8)	79 (± 4.8)	76 (± 5.6)	75 (± 6.0)
**New**	96 (± 2.0)	92 (± 4.0)	95 (± 2.8)	95 (± 3.2)

**Table 2.** Mean reaction times (ms) associated with correct responses to each item type separated by Retrieval Task, Switch/Stay and Group (Stroop/control). 95% confidence intervals in parentheses.

	Control		Stroop	
	Switch	Stay	Switch	Stay
***Function***				
** Target**	1018 (± 140)	962 (± 140)	1090 (± 120)	1057 (± 150)
** Nontarget**	1273 (± 200)	1120 (± 190)	1187 (± 130)	1156 (± 120)
** New**	843 ± (120)	819 (± 140)	850 (± 130)	756 (± 72)
***Drawing***				
** Target**	1010 (± 120)	1034 (± 180)	1067 (± 190)	975 (± 89)
** Nontarget**	1150 (± 150)	1106 (± 130)	1143 (± 110)	1183 (± 150)
** New**	785 (± 120)	789 (± 120)	768 (± 94)	736 (± 96)

The ANOVA conducted on accuracy data revealed main effects of Retrieval Task [ *F*_(1,46) _= 14.25, *p* < 0.001, η² _p_ = 0.24] and Item Type [ *F*_(1.7,77.0)_ = 59.03, *p* < 0.001, η² _p_ = 0.58] and a number of interactions, the highest order of which was Group x Switch/Stay x Retrieval Task x Item Type [ *F*_(1.8,82.9)_ = 3.38, *p* = 0.044, η² _p_ = 0.07]. These interactions reflected differential effects of the experimental factors of interest on accuracy to each item type; whereas target accuracy was not influenced by any factor (all *F*’s < 1), accuracy to new items was significant higher on switch than on stay trials [ *F*_(1,46)_ = 7.12, *p* = 0.01, η² _p_ = 0.13]. Nontarget accuracy showed a more complex pattern, with main effects observed for Switch/Stay [ *F*_(1,46)_ = 6.05, *p* = 0.018, η² _p_ = 0.12] and Retrieval Task [ *F*_(1,46)_ = 11.51, *p* = 0.001, η² _p_ = 0.20] both of which were moderated by group (Group x Switch/Stay; [ *F*_(1,46)_ = 12.17, *p* = 0.001, η² _p_ = 0.21], Group x Retrieval Task [ *F*_(1,46)_ = 4.55, *p* = 0.038, η² _p_ = 0.09]). The first of these interactions arose because nontarget accuracy increased from switch to stay trials for control participants only [ *F*_(1,23)_ = 16.06, *p* = 0.001, ωp² = 0.38]. The Group x Retrieval Task interaction reflected greater nontarget accuracy in the Function than the Draw retrieval task for the Stroop group only [ *F*_(1,23)_ = 12.38, *p* = 0.002, ωp² = 0.31].

The ANOVA conducted on RTs associated with accurate responses revealed main effects of Switch/Stay [ *F*_(1,46)_ = 9.43, *p* = 0.004, η² _p_ = 0.17] and Item Type [ *F*_(1.7,80.5)_ = 121.80, *p* < 0.001, η² _p_ = 0.73]. The former reflected faster responses on stay trials. Pairwise comparisons revealed that nontarget responses were slower than target responses [Item Type: *F*_(1,46)_ = 49.31, *p* < 0.001, η² _p_ = 0.52], which were in turn slower than new item responses [Item Type: *F*_(1,46)_ = 93.19, *p* < 0.001, η² _p_ = 0.67].

### ERP analyses

**Figure 1.**
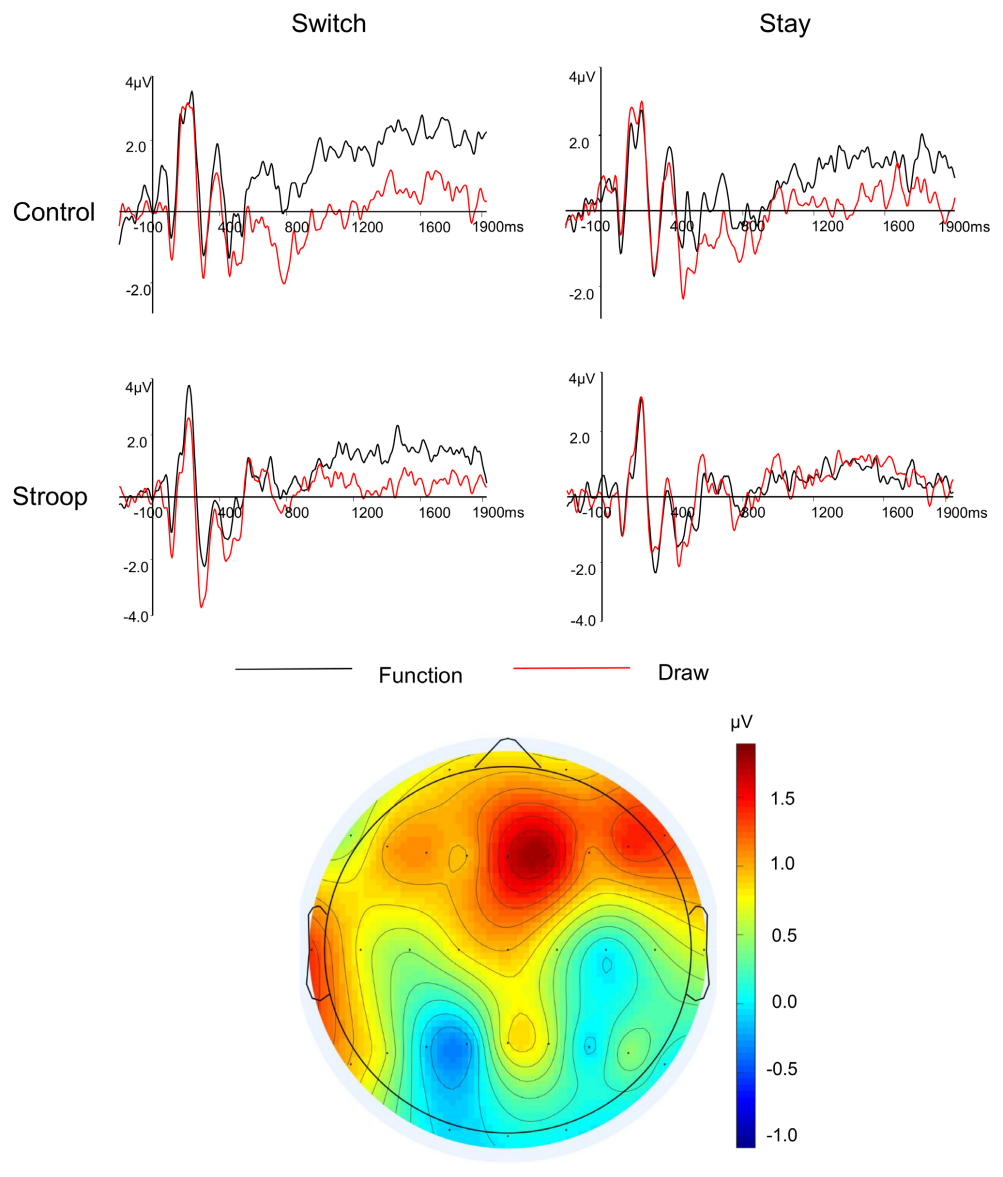
Upper: Pre-stimulus ERPs associated with Function and Draw cues at the superior right frontal electrode site (F2), separated by switch/stay trial status and group (Stroop/control). Amplitude (µV) is plotted on the y-axis across time (ms) on the x-axis. Lower: Voltage map shows the scalp distribution of the Function-Draw preparatory ERP effect observed on switch trials in the control group (scale bar indicates magnitude of effect in µV) between 700-1900ms, maximal in amplitude at F2.

***Pre-stimulus ERPs.*** Cue-locked ERPs preceding correct responses to all test items were averaged and separated according to retrieval task (function/draw), switch/stay trial status and group (Stroop/control). Figure 1 shows that frontal ERPs elicited by the function cue were more positive going than ERPs elicited by the draw cue. This effect was visually larger in magnitude in control than Stroop participants, and larger on switch than on stay trials.

The mean numbers of trials (minimum-maximum) forming averaged ERPs for each cue type were; control function switch: 48 (32-58), control function stay: 49 (33-56), control draw switch: 47 (32-55), control draw stay: 48 (37-55), Stroop function switch: 49 (43-56), Stroop function stay: 49 (41-58), Stroop draw switch: 48 (38-57), Stroop draw stay: 48 (39-58). Preparatory ERP effects of cue-type have previously been reported between 700–1900ms ^3^. The analysis of cue-related ERPs therefore included mean amplitude measurements between 700–1900ms from 24 electrode sites (F7/F8, F5/F6, F3/F4, F1/F2, T7/T8, C5/C6, C3/C4, C1/C2, P7/P8, P5/P6, P3/P4, P1/P2). The global mixed-model ANOVA incorporated within-subjects factors of Retrieval Task (function/draw), Switch/Stay, Anterior/Central/Posterior, Hemisphere and Site (inferior/mid-lateral/superior/midline) and one between-subjects factor of Group (Stroop/control). Significant effects involving Group were followed by repeated measures ANOVAs within each group.

The global ANOVA revealed interactions between Group x Retrieval Task x Site [ *F*_(2.2,103.0)_ = 3.57, *p* = 0.027, η² _p_ = 0.07] and Retrieval Task x Hemisphere x Site [ *F*_(2.9,132.1)_ = 2.96, *p* = 0.037, η² _p_ = 0.06]. The repeated measures ANOVA of control group data revealed a Retrieval Task x Anterior/Posterior interaction [ *F*_(1.5,34.6)_ = 5.80, *p* = 0.012, ωp² = 0.16], reflecting greater positivity for Function than Draw ERPs maximal at frontal sites. A Switch/Stay x Retrieval Task x Anterior/Posterior x Hemisphere interaction [ *F*_(1.7,39.4)_ = 4.07, *p* = 0.030, ωp² = 0.11] obtained in the same analysis confirmed that this effect was larger in magnitude on switch trials where the effect was largest in magnitude at right frontal electrode sites (see Figure 1). Post-hoc analysis of control group ERPs at frontal electrode sites confirmed a main effect of Retrieval Task on switch trials [ *F*_(1,23)_ = 6.50, *p* = 0.018, ωp² = 0.18] but not on stay trials. The repeated measures ANOVA of Stroop data indicated no effect of Retrieval Task, either in the global analysis of 24 electrode sites or when analysis was restricted to frontal sites. A follow-up group comparison conducted on subtraction Function-Draw ERPs at the frontal maxima (F3/F4, F2/F1, see Figure 1) of the pre-retrieval effect revealed a Group x Site interaction [ *F*_(1,46)_ = 4.82, *p* = 0.033, η² _p_ = 0.09], confirming a larger effect in the control group which increased in magnitude towards the midline.

**Figure 2.**
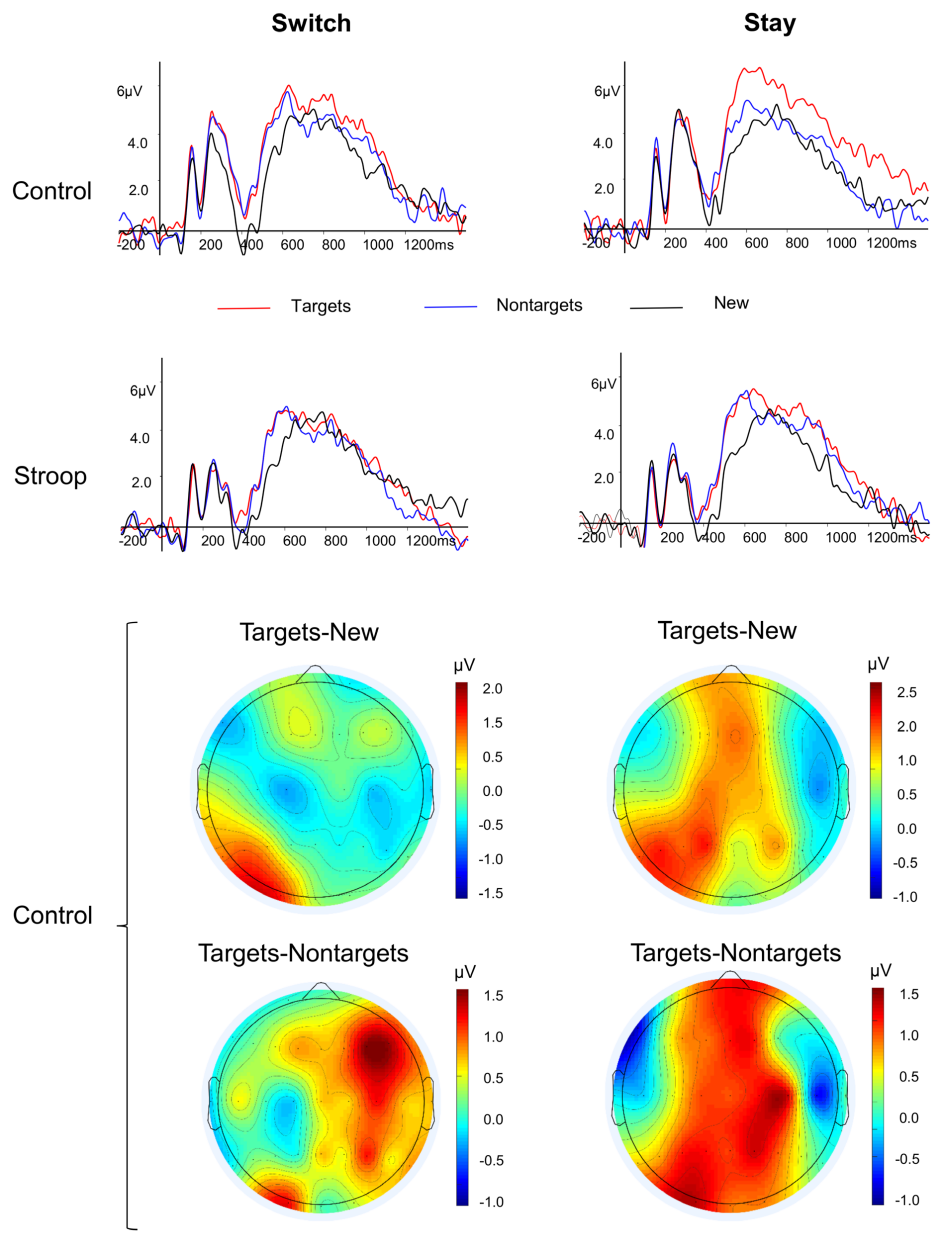
Upper: Item-locked ERPs associated with correctly classified targets, nontargets and new items at a left parietal electrode site (P3), separated by switch/stay trial status and group (Stroop/control). Amplitude (µV) is plotted on the y-axis across time (ms) on the x-axis. Lower: Voltage maps show the scalp distributions of the 500–800ms Target-New and Target-Nontarget ERP effects observed on switch and on stay trials in the control group (scale bars indicate magnitude of effect in µV).

***Item ERPs.*** Item-related ERPs were formed for correctly classified targets, nontargets and new items, separated by switch/stay trial status and group (Stroop/control). In order to obtain ERPs with good signal-to-noise, each item-related ERP was a weighted average of ERPs from both retrieval tasks. Figure 2 shows that target ERPs at left parietal electrode sites were more positive going than nontarget and new item ERPs on stay trials in the control group, and that this target prioritisation was not visually evident to the same degree on switch trials or for Stroop participants.

The mean numbers of trials (minimum-maximum) forming control group ERPs were; switch target: 31 (22-39), switch nontarget: 30 (18-37), switch new: 37 (29-40), stay target: 31 (24-37), stay nontarget: 31 (19-39), stay new: 36 (28-40). Trial numbers in the Stroop group were; switch target: 32 (21-37), switch nontarget: 31 (19-38), switch new: 37 (31-40), stay target: 32 (23-40), stay nontarget: 31 (16-38), stay new: 36 (29-40). As ERP measures of strategic retrieval have previously been obtained between 500-800ms ^22, 35, 47^, analysis of item-related ERPs incorporated mean amplitude measurements during this time window from the same 24 electrode sites described above. The global ANOVA incorporated within-subjects factors of Item Type (target/nontarget/new), Switch/Stay, Anterior/Central/Posterior, Hemisphere, and Site (inferior/mid-lateral/superior/midline) and one between-subjects factor of Group (Stroop/control). Effects of Item Type (Target/Nontarget/New) were followed up with pairwise comparisons (see Table 3). Once constrained to pairs of experimental conditions, mixed-model ANOVAs revealing significant effects of Group were followed up with repeated measures ANOVAs within each group.

**Table 3.** Results ( *p* < 0.05) of mixed model ANOVAs conducted on stimulus-locked ERPs associated with correct responses to each pair of item types (Target/Nontarget/New) during the 500-800ms latency region.

	Target/Nontarget	Target/New	Nontarget/New
IT	*F*_(1,46)_ = 6.57, *p* = 0.014, η² _p_ = 0.15		
IT x HM		*F*_(1,46)_ = 10.94, *p* = 0.002, η² _p_ = 0.19	*F*_(1,46)_ = 15.49, *p* < 0.001, η² _p_ = 0.25
IT x ST	*F*_(1.7,78.2)_ = 5.03, *p* = 0.012, η² _p_ = 0.10	*F*_(1.6,74.4)_ = 6.67, *p* = 0.004, η² _p_ = 0.13	
GP x SW x HM		*F _(1,46)_* = 6.04, *p* = 0.018, η² _p_ = 0.13	
IT x AP x HM		*F*_(1.8,84.8)_ = 4.69, *p* = 0.014, η² _p_ = 0.09	*F*_(1.8,83.8)_ = 5.42, *p* = 0.008, η² _p_ = 0.12
IT x AP x ST		*F*_(4.0,185.4)_ = 2.87, *p* = 0.024, η² _p_ = 0.06	
IT x HM x ST		*F*_(2.4,112.0)_ = 6.82, *p* = 0.001, η² _p_ = 0.13	*F*_(2.6,120.3)_ = 5.56, *p* = 0.002, η² _p_ = 0.11
GP x SW x IT x ST	*F*_(1.9,89.2)_ = 4.16, *p* = 0.020, η² _p_ = 0.07	*F*_(1.7,79.4)_ = 4.51, *p* = 0.018, η² _p_ = 0.09	
SW x IT x AP x HM x ST		*F*_(5.0,230.5)_ = 2.94, *p* = 0.014, η² _p_ = 0.06	

GP = Group (Stroop/control), SW = Switch/Stay, IT = Item Type, HM = Hemisphere, AP = Anterior/Central/Posterior, ST = Site (inferior/mid-lateral/superior/midline).

The global analysis revealed a number of interactions involving Item Type (Item Type x Hemisphere [ *F*_(1.9,85.3)_ = 10.19, *p* < 0.001, η² _p_ = 0.18], Item Type x Site [ *F*_(3.3,149.6)_ = 4.28, *p* = 0.005, η² _p_ = 0.09], Item Type x Anterior/Posterior x Hemisphere [ *F*_(3.1,141.9)_ = 3.91, *p* = 0.010, η² _p_ = 0.08], Item Type x Hemisphere x Site [ *F*_(4.7,217.1)_ = 5.08, *p* < 0.001, η² _p_ = 0.10]) as well as higher level interactions between Group x Switch/Stay x Item Type x Site [ *F*_(3.5,160.2)_ = 3.07, *p* = 0.023, η² _p_ = 0.06] and Switch/Stay x Item Type x Anterior/Posterior x Hemisphere x Site [ *F*_(8.8,404.2)_ = 1.98, *p* = 0.042, η² _p_ = 0.04]. The pairwise comparison of targets and new items revealed significant effects of Item Type including Group x Switch/Stay x Item Type x Site (see Table 3). Repeated measures ANOVA of control targets/new items revealed Item Type x Anterior/Posterior x Hemisphere [ *F*_(2.0,45.3)_ = 6.85, *p* = 0.003, ωp² = 0.20] and Switch-Stay x Item Type x Site [ *F*_(2.0,46.1)_ = 3.98, *p* = 0.025, ωp² = 0.11] interactions. These reflected greater positivity for targets with a left posterior focus which increased in magnitude from switch to stay trials (see Figure 2). Post-hoc analyses indicated significant effects of Item Type both on switch trials (Item Type x Anterior/Posterior x Hemisphere x Site: [ *F*_(4.5,103.5)_ = 2.89, *p* = 0.021, ωp² = 0.07]) and on stay trials (Item Type x Site: [ *F*_(1.8,40.5)_ = 5.05, *p* = 0.014, ωp² = 0.14], with main effects of Item Type at the left parietal maxima (switch: [ *F*_(1,23)_ = 4.36, *p* = 0.048, ωp² = 0.12], stay: [ *F*_(1,23)_ = 6.45, *p* = 0.018, ωp² = 0.18]). Repeated measures ANOVA of Stroop targets/new items revealed an Item Type x Hemisphere x Site interaction [ *F*_(2.3,53.5)_ = 4.13, *p* = 0.017, ωp² = 0.11]. This crossover interaction reflected a small positivity for targets at left hemisphere sites and an effect of reversed polarity at inferior right hemisphere sites, neither of which were significant in post-hoc analyses. A follow-up group comparison conducted on subtraction target-new ERPs at the left parietal maxima (see Figure 2) revealed a Group x Switch/Stay x Site interaction [ *F*_(1,46)_ = 5.59, *p* = 0.022, η² _p_ = 0.11], confirming a larger effect in the control group which increased in magnitude from switch to stay trials.

The pairwise comparison of targets and nontargets revealed effects of Item Type including Group x Switch/Stay x Item Type x Site (see Table 3). These reflected greater positivity for targets than nontargets, an effect that was largest in magnitude for control participants on stay trials (see Figure 2). Repeated measures ANOVA of control targets/nontargets revealed a main effect of Item Type [ *F*_(1,23)_ = 4.64, *p* = 0.042, ωp² = 0.13] and interactions between Item Type x Site [ *F*_(1.3,30.7)_ = 5.60, *p* = 0.017, ωp² = 0.15] and Switch/Stay x Item Type x Hemisphere [ *F*_(1,23)_ = 4.54, *p* = 0.044, ωp² = 0.12]. Post-hoc analyses indicated a right-lateralised positivity for targets on switch trials (Item Type: [ *F*_(1,23)_ = 5.65, *p* = 0.026, ωp² = 0.17], Item Type x Hemisphere: [ *F*_(1,23)_ = 7.19, *p* = 0.013, ωp² = 0.20]) and greater positivity for targets maximal towards the midline on stay trials (Item Type x Site [ *F*_(1.7,39.0)_ = 6.84, *p* = 0.004, ωp² = 0.19] (see Figure 2). No effects of item type were obtained in the repeated measures ANOVA of targets and nontargets in the Stroop group. A follow-up group comparison conducted on subtraction target-nontarget ERPs at the stay trial parietal maxima (P2) revealed a main effect of Group [ *F*_(1,46)_ = 5.39, *p* = 0.025, η² _p_ = 0.10], confirming a larger effect in the control group.

A topographic analysis of control group target/nontarget effects assessed whether different neural generators were recruited on switch and on stay trials. Difference scores (formed by subtracting nontarget from target mean amplitudes) were rescaled using the max-min method to remove changes in effect magnitude from changes in topography ^48^. A repeated measures ANOVA comprised the factors of Switch/Stay, Anterior/Posterior, Hemisphere and Site (inferior/mid-lateral/superior/midline). A Switch/Stay x Site interaction [ *F*_(2.0,45.3)_ = 3.36, *p* = 0.044, ωp² = 0.09] indicated that different neural generators gave rise to the distinct scalp distributions evident on switch and on stay trials (see Figure 2).

Finally, the pairwise comparison of nontargets and new items revealed several interactions involving Item Type (see Table 3), reflecting greater positivity for nontargets at left posterior inferior sites and greater positivity for new items maximal at right central superior sites. No moderating effects of Group or Switch/Stay were observed. Post-hoc analyses indicated that the positivity observed for nontargets maximal at P7 [ *F*_(1,46)_ = 9.31, *p* = 0.004, ωp² = 0.15] and the negativity observed for these items at right hemisphere sites [ *F*_(1,46)_ = 5.57, *p* = 0.023, ωp² = 0.09] were both statistically significant.

## Discussion

The impact of cognitive control reserves on ERP measures of goal-directed memory retrieval was examined via two experimental hypotheses. First, it was predicted that performing a Stroop task before the memory test would attenuate ERP measures of retrieval orientation initiation. Second, it was predicted that ERP measures of strategic retrieval (i.e. prioritised recollection of targets) in a task-switching design would be attenuated in participants who first completed the Stroop task. Both hypotheses were supported by the findings and will be discussed in turn.

A series of studies from our lab have demonstrated the sensitivity of ERPs to different episodic goals during the pre-stimulus interval ^2, 3, 11, 27^. We have also recently shown that this measure predicts the success or failure of criterial recollection ^5, 49^. Most of these studies have employed task switching designs in which different pre-stimulus cues signal the episodic content to be retrieved. The purpose of this design is to induce the initiation of different retrieval orientations many times within a single testing session, and ERPs typically diverge according to task goals when a new memory task begins (‘switch’ trials). Because these divergences are reduced on subsequent trials within the testing sequence, pre-stimulus ERP effects observed on switch trials have been attributed to processes that initiate retrieval orientations, but which are not required to sustain them across subsequent items within that task. Pre-stimulus ERPs obtained from the control group here are consistent with this account; preparatory ‘function’ ERPs showed a significant positive shift relative to ‘drawing’ ERPs on switch trials only. As in previous studies, this was a temporally sustained effect with a frontal distribution. The control group data therefore confirmed that the paradigm had captured the ERP index of retrieval orientation initiation obtained in previous studies. Importantly, this effect was attenuated - and not statistically significant - in the Stroop group. This extends the discovery that cognitive control depletion reduces ERP measures of retrieval orientation maintenance ^27^ to processes involved in their initiation. Both components of retrieval orientation are sensitive to reserves of cognitive control available during retrieval. It is important to note that this group effect was not mirrored in the target accuracy rates. Indeed, pre-retrieval correlates of orientation have not yet been linked with retrieval accuracy at the level of group or individual differences, even though they predict source memory accuracy at the item level (i.e. when pre-retrieval ERPs are back-sorted according to memory accuracy in within subject analyses). These “preparatory memory effects” are arguably unlikely to reflect simple modulations of attentional resources, as they differ qualitatively according to the nature of the contextual information to be retrieved ^5, 49^. However, the reason why pre-stimulus measures of retrieval orientation predict retrieval accuracy at the level of items but not participants is an important goal for future research.

The Stroop task also influenced neural correlates of strategic retrieval. The response requirements of the memory test used here are borrowed from the exclusion task ^34^; studied items from a specified encoding context require a positive response while those from the alternate context are ‘excluded’ on the same response key as new items. These response requirements encourage participants to treat items from the specified context as memory ‘targets’ and to constrain retrieval processing accordingly, and neural evidence for this comes in at least three different forms. First, neural activity associated with identical unstudied items differs between exclusion tasks with different retrieval goals (see Introduction). Second, pre-stimulus ERPs also differ according to target designation ^11, 27^. Third, ERP measures of recollection associated with nontargets are significantly smaller than those associated with targets, this being the parietal old/new effect in ERP studies ^12, 22, 35, 36^ and activation in angular gyrus in their fMRI counterpart ^18^. This third aspect is termed ‘strategic retrieval’ and ** there was evidence for this in control participants here, with target ERPs eliciting greater positivity than both nontargets and new items *.* Importantly, participants who completed the Stroop task showed no evidence of strategic retrieval on either switch or stay trials, as target and nontarget ERPs did not differ. This indicates that the combined cognitive load of task-switching and Stroop performance prevented these participants from engaging memory control processes necessary for flexible strategic retrieval. While it is important to remember that correctly classified nontarget ERPs are contaminated by forgetting in the exclusion paradigm, the finding that nontarget/new ERP effects were not affected by either the Stroop manipulation or the switch/stay trial status indicates that modulation of the strategic retrieval ERP effects were not driven by the differential suppression of nontarget recollection. These factors instead influenced the target/new ERP effects, indicating that the modulation of strategic retrieval was instead driven by differences in target recollection.

Elward *et al.* ^33^ also found that ERP correlates of strategic retrieval observed in participants with high working memory capacity were eliminated following the Stroop task, even when retrieval tasks were blocked. This finding contrasted with a second study ^27^, which found ERP evidence of strategic retrieval in the same pair of blocked memory tasks following Stroop performance. The cause for this discrepancy may lie in the overall amount of cognitive depletion. Elward *et al.*’s participants completed the O-span test of working memory capacity in addition to the Stroop task before encoding, which may have imposed a further cognitive load. The present study administered the Stroop task between study and test, but the convergence of the present findings with those of Elward *et al.* suggests that the point at which the Stroop task was administered was less important than the degree to which reserves of cognitive control were taxed by other aspects of the experiment. Here, requiring participants to attend to pre-stimulus cues and switch between retrieval tasks increased the cognitive load during retrieval. It is likely that this made strategic retrieval more vulnerable to disruption when reserves of cognitive control were depleted by the Stroop task than in the blocked paradigm employed previously ^27^.

The control group data also showed that task-switching influences strategic retrieval more generally, as the topographies of the target/nontarget effects differed between switch and stay trials. The switch trial effect was focused over right frontal electrode sites whereas the stay trial effect was larger at temporal and parietal sites. This is perhaps surprising given that ERP correlates of retrieval orientation were evident on switch trials for this group. One interpretation is that there is a delay between the initiation of a retrieval orientation and the effective operation of downstream processes that support parietally distributed strategic retrieval ERP effects. An alternative account is that the two are not intrinsically linked; that strategic retrieval is not always a consequence of – and does not necessarily require – a goal-directed memory state. For example, it has been proposed that strategic retrieval can arise either as a consequence of top-down cognitive control processes (such as proactive retrieval orientations) or from bottom-up processes such as the ease of access to nontarget representations ^22, 50^. Evidence for this second view comes from the blocked version of this study ^27^, where correlates of strategic retrieval were evident for the Stroop group in the absence of correlates of retrieval orientation. These participants showed enhanced ERP measures of post-retrieval monitoring at right frontal sites relative to controls, indicating that reactive control was used to compensate for reduced proactive control. The right frontal distribution of the target/nontarget effect on switch trials in the present study suggests that this may also have occurred here, but that post-retrieval monitoring was not required to the same degree on stay trials once target recollection was prioritised at the point of retrieval.

Levels of memory accuracy and associated reaction times for targets were not affected by the Stroop/control manipulation. This replicates previous studies that found equivalent levels of memory accuracy for Stroop and control participants ^27, 33^. It is notable that memory responses in all three studies were self-paced. Imposing a response deadline could potentially decrease memory accuracy in Stroop participants, as this would limit their opportunity to engage post-retrieval monitoring processes. It is also possible that cognitive control depletion results in more nuanced memory deficits that cannot be detected with relatively blunt measures such as accuracy and RT, such as the qualitative details of memories. The task-switching manipulation did introduce subtle differences in response accuracy between the two groups of participants. Nontarget accuracy increased from switch to stay trials in the control group, and this behavioural shift was accompanied by the emergence of parietal target/nontarget ERP effects on stay trials. Strategic retrieval was therefore associated with an improved ability to exclude nontargets rather than to identify targets for this group. This behavioural shift in nontarget accuracy was absent in Stroop participants. These data also potentially speak to the current controversy regarding the validity of cognitive control (or ‘ego’) depletion as a construct. Despite the large and high-profile ego depletion literature, certain meta-analyses and registered reports have failed to replicate the effect. In a recent review, Friese *et al.* (2019) noted that the nature of both the independent variable (i.e. the depletion manipulation) and the dependent variable (i.e. measures taken during the post-depletion task) have been highly variable across the literature. The depletion manipulation can vary in intensity and can also be cumulative across an experimental session, as discussed above in terms of cognitive load. In the present study, the post-depletion task (the task-switching source memory task) was designed to maximize sensitivity to depleted levels of cognitive control. Nonetheless, it is notable that the sensitivity of the behavioural and the electrophysiological dependent measures were not equivalent, with depletion effects being far more substantial in the latter. These findings suggest that future studies of ego depletion may benefit from examining neural and physiological correlates of task performance as a well as behavioural measures ^51^.

In conclusion, pre-stimulus ERP correlates of retrieval orientation initiation were observed on switch trials in a control group and eliminated in a group of participants who first completed the Stroop task. Similarly, established ERP correlates of strategic retrieval (enhanced correlates of recollection for memory targets versus nontargets) were evident in the control group and eliminated in the Stroop group. Task-switching at test also influenced strategic retrieval processing, with ERP correlates of target versus nontarget recollection shifting from a frontal to a temporo-parietal distribution for control group participants. In combination, the ERP data indicate that depleting reserves of cognitive control impairs the ability to modulate strategic memory processing in response to changing retrieval goals.

## Data availability

### Underlying data

Open Science Framework: Cognitive control depletion reduces pre-stimulus and recollection-related measures of strategic retrieval. https://doi.org/10.17605/OSF.IO/5KURQ ^46^

This project contains the following underlying data:

- Behavioural data (raw behavioural data obtained for each participant, provided as *.csv files, accuracy codes are specified in the outcodes.txt file)
- ERP files (event-related potentials for each participant, provided as *.erp files)
- Raw EEG data (raw EEG data for each participant, provided as *.bdf files)
- The ERP analysis programme ALASBIG.exe and existing documentation

Data are available under the terms of the Creative Commons Zero "No rights reserved" data waiver (CC0 1.0 Public domain dedication).
