# Supplementary material for: Cognitive control depletion reduces pre-stimulus and recollection-related measures of strategic retrieval
Source: Wellcome Open Res. 2019 Oct 18;4:120. Originally published 2019 Aug 13. [Version 2] doi: 10.12688/wellcomeopenres.15347.2 (PMC6737995; doi:10.12688/wellcomeopenres.15347.2)
Supplement: Peer review history

# Peer review: Reviewer response for version 2

**DOI:** 10.21956/wellcomeopenres.16978.r36811

**Published:** 2019-11-06

**Authors:** Joshua Koen

**Affiliations:** 1 Department of Psychology, University of Notre Dame, Notre Dame, IN, USA

The author addressed all of my major concerns from my initial review. Although I do think my prior comment regarding distinguishing recollection and familiarity might explain some of the null behavioral results, I believe this issue is better addressed in future research. The revisions have improved what was already a strong experiment and paper.   

I confirm that I have read this submission and believe that I have an appropriate level of expertise to confirm that it is of an acceptable scientific standard.

# Peer review: Reviewer response for version 2

**DOI:** 10.21956/wellcomeopenres.16978.r36812

**Published:** 2019-10-28

**Authors:** Timm Rosburg

**Affiliations:** 1 Forensic Department, University Psychiatric Clinics Basel, Basel, Switzerland

The author addressed the issues I have raised in my previous comments. However, I might note that much of her interpretation of the findings is still based on an assumption, namely that the Stroop task leads to a state conceptualized as ego depletion, as also indicated by the title of the study. This assumption might not turn out to be true, as previously mentioned. There might not even be such thing as ego depletion, as recently reviewed by Friese and co-workers ^1^. Among others, Job and co-workers showed in their study that individuals who viewed the cognitive control capacity as not limited did not show behavioral effects of ego depletion tasks ^2^.

I confirm that I have read this submission and believe that I have an appropriate level of expertise to confirm that it is of an acceptable scientific standard.

## References

[1] : Is Ego Depletion Real? An Analysis of Arguments. *Pers Soc Psychol Rev*.23(2) : 10.1177/1088868318762183 107-131 10.1177/1088868318762183 29591537

[2] : Ego depletion--is it all in your head? implicit theories about willpower affect self-regulation. *Psychol Sci*.2010;21(11) : 10.1177/0956797610384745 1686-93 10.1177/0956797610384745 20876879

# Peer review: Reviewer response for version 2

**DOI:** 10.21956/wellcomeopenres.16978.r36813

**Published:** 2019-10-21

**Authors:** Rachael Elward

**Affiliations:** 1 London South Bank University, London, UK

The authors have addressed the concerns that I raised in my previous report. I have no additional comments.

I confirm that I have read this submission and believe that I have an appropriate level of expertise to confirm that it is of an acceptable scientific standard.

# Peer review: Reviewer response for version 1

**DOI:** 10.21956/wellcomeopenres.16759.r36335

**Published:** 2019-10-14

**Authors:** Joshua Koen

**Affiliations:** 1 Department of Psychology, University of Notre Dame, Notre Dame, IN, USA

This paper reports an ERP study examining the impact of cognitive control depletion on two retrieval processes associated with retrieval of task-relevant contextual details: retrieval orientation and strategic retrieval. The author predicted that depletion of cognitive control resources would eliminate ERP correlates of retrieval orientation and strategic retrieval. Two groups of participants studied words while making one of two semantic judgments for a subsequent exclusion-based recognition test. Following the encoding phase, one group of participants completed a Stroop task to deplete cognitive control resources. The control group completed a simple reading task. The exclusion memory test adopted a local task-switching approach whereby participants were given a pre-stimulus cue indicating which condition (i.e., encoding task) was the target mnemonic information on a given trial. The test was setup such that memory performance and ERPs could be examined for stay trials and switch trials. The results were largely consistent with the hypothesis. The control group showed significant ERP pre-stimulus retrieval orientation effects (on switch trials) and a larger left parietal old/new effect for correctly judged target versus non-target items (on stay trials). This latter finding is evidence of prioritized (or strategic) retrieval. These effects were reported absent in the Stroop group.

 This is a well-designed study that makes a significant contribution to the field. The methods and analysis are sound and sufficient information is provided to replicate the methods (and the raw data are also available).

 I have a few minor comments:

1. I was rather surprised that memory was not affected in any appreciable way by the Stroop manipulation. The hypothesis driving much of this research is that cognitive control depletion will negatively affect retrieval processes associated with recollection. I do wonder if part of this null effect might be driven by not providing separate estimates of recollection and familiarity. The accuracy measures reported do not necessarily isolate (i.e., exclusion tests are not process pure), and thus the contribution of familiarity might be masking effects.
2. One of the curious behavioural findings was the interaction showing improved exclusion of non-targets from switch to stay trials in the Control group, but not in the Stroop group. I found it surprising that, in the Control group, exclusion of non-targets on Switch trials were *lower *(.75) than Stay trials (.85) and both Switch and Stay trials in the Stroop group (.84 for both). This result does not seem to fit with the main conclusions that depletion of cognitive control resources reduces strategic retrieval. Instead, it seems to suggest that Stroop participants are better able to reject non-targets on Switch trials and indicates cognitive control depletion might benefit performance somehow (one possibility is a trade off between recollection and familiarity). Admittedly, this is finding is only specific to one of the tasks, so it might not be that important (which, if this is the case, discussion of this result should be de-emphasized in the conclusion).
3. The post-hoc tests for interactions involving group never directly compared groups. Instead, these tests only examined if memory-related effects were significant *within* a group. It is unclear if the lack of significance in the Stroop group reflects a significantly reduced effect relative to the Control group. I would have liked to see this comparison. To be clear, I do not believe any of the main conclusions would be undermined by results from this comparison, but just might require a little more nuance. It also might be useful to have a figure plotting the target-new and target-non-target effects from the 500-800ms time window from P3 (or another representative electrode). This would provide some sense of variability to readers, which is currently difficult to get at.
4. I also would have liked to see the ERP results for the earlier 300-500ms time window that is typically associated with familiarity (i.e., the FN400). I understand the focus of the left parietal old/new effect given the hypothesized role of cognitive control and the retrieval processes being studied on recollection (both here and in many prior studies). However, this does not mean that such reductions in resources might not also be present for familiarity. This analysis might shed some light on if participants in the Control group relied more on familiarity.

I confirm that I have read this submission and believe that I have an appropriate level of expertise to confirm that it is of an acceptable scientific standard, however I have significant reservations, as outlined above.

# Response

**Published:** 2019-10-17

**Authors:** Jane E. Herron

**Affiliations:** Cardiff University, UK

Thank you for your constructive feedback. I had already submitted version 2 before receiving your review, but hopefully some of your comments have been addressed in this new version. For example, between group contrasts of the effects of interest (subtraction waveforms) have now been added to the manuscript as this was raised by another reviewer. Figure 2 shows the target, nontarget and new ERPs at P3 together with scalp maps of the target-new and target-nontarget effects between 500-800ms; is there further data that would be helpful to see? Thank you for your interesting comments about potential contributions of familiarity to performance, I will take a look at the ERP effects between 300-500ms before submitting a further version. While I have extended the discussion of the insensitivity of memory accuracy to the Stroop manipulation in version 2, I hadn't considered that familiarity and recollection may be affected differently, and it will be interesting to see how the 300-500ms ERP data informs that possibility.

# Peer review: Reviewer response for version 1

**DOI:** 10.21956/wellcomeopenres.16759.r36337

**Published:** 2019-09-10

**Authors:** Timm Rosburg

**Affiliations:** 1 Forensic Department, University Psychiatric Clinics Basel, Basel, Switzerland

The event-related potential (ERP) study of Herron addresses the question how two forms of strategic retrieval (prioritization of target over nontarget retrieval, as well as initiation of retrieval orientation) are affected by ego depletion. Ego depletion (or cognitive control depletion) refers to the idea that volitional acts draw on a limited resource and, by this, effortful acts of cognitive control have some detrimental effect on the performance in subsequent tasks ^1^. In the study of Herron, participants encoded object words in two conditions. After this, participants performed either a cognitively demanding Stroop task or, as control condition, an easy reading task. At test, participants performed a memory exclusion task, with pre-stimulus retrieval cues indicating which category of old items had to be considered as targets. Old items of the second study condition had to be rejected as nontargets, together with newly presented items. Importantly, the designated target category switched after two test items each (resulting in one switch and one stay trial in succession). In the control condition, participants showed a pre-stimulus retrieval orientation effect in switch trials and a larger left-parietal old/new effect for targets than for nontargets in stay trials. Both effects were abolished after ego depletion. Herron concludes that both forms of strategic retrieval are susceptible to cognitive control depletion.

 Overall, the study is well-designed and state-of-the-art. Moreover, the study nicely complements previous studies in the field. With regard to the technical qualities of the study, I only have few remarks. With regard to the significance of the findings, I would like to make some comments, which are intended as thought-provoking impulse rather than as categorial critique. These comments primarily concern the presumed neurocognitive correlates of strategic retrieval.

*Left-parietal old/new effect and strategic retrieval*

 Strategic retrieval has been defined as cognitive control processes initiated in order to optimize retrieval success, as well as to minimize retrieval effort ^2^. The prioritization of target information over nontarget information has been considered as one form of strategic retrieval (initially discussed in ^3^). Target prioritization may manifest in a significantly larger left-parietal old/new effect for target information than for nontarget information, as outlined by Herron. One problem with this definition is that ERPs in episodic memory studies are calculated on the basis of correct responses, i.e. hits for targets and correct rejections (CRs) for nontargets. However, in memory exclusion tasks, the CR rate for nontargets is usually larger than the hit rate ^4^^,^^5^) because the CR rate is inflated by the percentage of nontargets that have been forgotten. Such trials would be included in calculating the ERPs and lead to some diminishment of the left-parietal old/new effect as correlate of recollection for nontargets, as compared to targets. In an alternative approach, target prioritization has been defined as the absence of a left-parietal old/new effect for nontargets ^6^. An absent left-parietal old/new effect for nontargets suggests that source information of nontargets has not been retrieved and the target identification is primarily based on the retrieval of target information (= target prioritization) and not on a recall-to-reject strategy ^7^. This operationalization is not perfect either as the absence of nontarget effects might be due to the lack of statistical power.

 Notwithstanding this methodological problem, let us assume that at the memory exclusion test participants retrieve to some extent nontarget information in addition to target information, because they actively seek to retrieve the nontarget information or maybe simply because this nontarget information is easily accessible and comes to mind, when encountering the nontarget retrieval cue ^8^. What would be the consequences when a participant seeks to prioritize target information and override this kind of volitional or incidental recollection ^9^? - If successful, the consequences of such strategical retrieval should be seen for the left parietal old/new effect for targets *and* for nontargets because a more extensive retrieval of target information should make the retrieval of nontarget information unnecessary. This pattern has, however, not been observed in the current study. The left-parietal old/new effect for nontargets did vary neither between trials (switch vs. stay) nor between groups (ego depletion vs. control). Both factors selectively affected target retrieval: A larger left-parietal old/new effect for targets than for nontargets was only present at stay trials in the control condition. This might suggest that switching to a new target category as well as having performed a cognitive demanding task beforehand diminishes the resources available for target identification and target retrieval rather than affecting strategic retrieval. Importantly, similar effects of task switching on the left-parietal old/new effect have previously been observed in recognition and source memory tasks ^10^^,^^11^. Impaired retrieval of target information in switch trials or following an ego depletion task might affect target prioritization, but the current findings do, from my point of view, not provide evidence for an impaired prioritization of target information after ego depletion.

*Retrieval orientation and strategic retrieval*

 For post-stimulus ERPs, the benefits of adopting an appropriate retrieval orientation are empirically relatively well validated and also highly plausible ^4^^,^^12^^,^^13^^,^^14^^,^^15^. One could argue that the retrieval orientation effect reflects, to some extent, the preparedness of a rememberer for retrieving the targeted episodic information. For pre-stimulus ERPs, the observed retrieval orientation effects are not easy to conceptualize: In one study ^16^, task preparatory pre-stimulus ERP effects were evident in switch but not in stay trials, similar to the current study. One possible cause for this non-observation could be that sustained activity is difficult to detect by conventional ERP recordings ^16^. In another study ^17^, it was found that the task preparatory pre-stimulus ERP effects predicted subsequent retrieval success. In a further follow-up study, the pre-stimulus retrieval orientation effects varied in polarity between switch and stay trials, whereby only the retrieval orientation effects in switch trials showed an association with retrieval accuracy ^18^. The authors linked the pre-stimulus effect in switch trials to the initiation of retrieval orientation operations, whereas the pre-stimulus effect in stay trials was linked to the maintenance of retrieval orientation operations.

 The current study does not report on the association between pre-stimulus retrieval orientation effects and retrieval accuracy, but it appears that the pre-stimulus retrieval orientation effect and the left-parietal old/new effect did not co-vary because the largest retrieval orientation effect was observed in switch trials of the control condition, whereas the largest left-parietal old/new effect was observed in stay trials of the control condition. Moreover, the retrieval accuracy was the same in the two groups even though the retrieval orientation effect varied between them. Thus, the suppression of the retrieval orientation effect had apparently no consequence on the retrieval accuracy.

 A maybe more fundamental question is whether retrieval orientation can be considered as form of strategic retrieval or whether it reflects, as proposed above, more or less just the preparedness of a rememberer for retrieving the targeted episodic information. The term ‘strategic’ implies that there is some cognitive process contributing to the retrieval outcome, which is not necessarily in place by default. The term also suggests that this process could be instructed or trained and would lead to some improvement in retrieval accuracy. I am aware of just one study which instructed participants to prioritize target information at retrieval ^19^. However, this study revealed no impact of instruction on retrieval accuracy (as compared to another group not receiving such instruction). The ERP data revealed no evidence for an effectiveness of this instruction either. Thus, the empirical evidence for retrieval orientation as process of strategic retrieval is, at best, scarce. This principle issue requires some further research and, from my point of view, one should be cautious to label retrieval orientation as ‘strategic’ pre-retrieval process.

*Stroop as ego depletion task*

 The effects of the task (Stroop vs. control) on the ERPs are surprisingly large. What does it mean for the work as scientist, if 6.5 min of a cognitively demanding task already have such a strong detrimental effect on cognition? Should scientists be more careful when they spend their time racking their brains to solve the world´s mysteries? – Apparently, the 6.5 min of the Stroop task had some considerable impact on the subsequent retrieval processes. However, again I would encourage to be more cautious to attribute the consequences of the task to ego depletion because the fact that the task was intended for this purpose does not mean that ego depletion is the cause for its consequences. Among others, such a task could modulate arousal and motivation.

*Some technical notes*

 Overall, the paper is well written. However, the author might consider the following issues: (a) The thresholds for the artifact detection are not specified and should be added. (b) The ALAS software tool is not further specified and no reference is provided. Some additional information would be helpful. (c) In Figure 1, the scaling of the ordinate should be 2.0 (and not 2.1). Moreover, it should be µV (and not µv). (d) I would recommend to use a different color scaling for the voltage map in Figure 1. Blue colors should indicate negative and red colors positive voltage values. In its current form, the color intensity provides the initial impression that the negative and positive amplitude values were equally large. Moreover, the time range needs to be indicated.

I confirm that I have read this submission and believe that I have an appropriate level of expertise to confirm that it is of an acceptable scientific standard, however I have significant reservations, as outlined above.

## References

[1] : Ego depletion: Is the active self a limited resource?. *Journal of Personality and Social Psychology*.1998;74(5) : 10.1037//0022-3514.74.5.1252 1252-1265 10.1037//0022-3514.74.5.1252 9599441

[2] : Strategic retrieval and retrieval orientation in reality monitoring studied by event-related potentials (ERPs). *Neuropsychologia*.2013;51(3) : 10.1016/j.neuropsychologia.2012.11.014 557-71 10.1016/j.neuropsychologia.2012.11.014 23168131

[3] : Strategic influences on recollection in the exclusion task: Electrophysiological evidence. *Psychonomic Bulletin & Review*.2003;10(3) : 10.3758/BF03196535 703-710 10.3758/BF03196535 14620367

[4] : Neural correlates of individual differences in strategic retrieval processing. *J Exp Psychol Learn Mem Cogn*.2009;35(5) : 10.1037/a0016375 1175-86 10.1037/a0016375 19686013

[5] : Electrophysiological correlates of retrieval orientation in reality monitoring. *Neuroimage*.2011;54(4) : 10.1016/j.neuroimage.2010.10.068 3076-84 10.1016/j.neuroimage.2010.10.068 21044886

[6] : The costs of target prioritization and the external requirements for using a recall-to-reject strategy in memory exclusion tasks: a meta-analysis. *Psychon Bull Rev*.2017;24(6) : 10.3758/s13423-017-1256-1 1844-1855 10.3758/s13423-017-1256-1 28299598

[7] : Word frequency effects in associative and item recognition. *Mem Cognit*.1992;20(3) : 10.3758/bf03199660 231-43 10.3758/bf03199660 1508049

[8] : Strategic retrieval in a reality monitoring task. *Neuropsychologia*.2011;49(10) : 10.1016/j.neuropsychologia.2011.07.002 2957-69 10.1016/j.neuropsychologia.2011.07.002 21763332

[9] : Retrieval volition and memorial awareness in stem completion: an empirical analysis. *Psychol Res*.1995;57(3-4) :166-78 775394710.1007/BF00431278

[10] : Effects of task-set adoption on ERP correlates of controlled and automatic recognition memory. *Neuroimage*.2011;55(3) : 10.1016/j.neuroimage.2010.12.059 1384-92 10.1016/j.neuroimage.2010.12.059 21211568PMC3049819

[11] : Electrophysiological insights into control over recollection. *Cogn Neurosci*.2012;3(3-4) : 10.1080/17588928.2012.662217 168-73 10.1080/17588928.2012.662217 24171734

[12] : Electrophysiologically dissociating episodic preretrieval processing. *J Cogn Neurosci*.2012;24(6) : 10.1162/jocn_a_00152 1476-91 10.1162/jocn_a_00152 21981675

[13] : Timing matters: age-related changes in episodic retrieval control as revealed by event-related potentials. *Brain Res*.2013;1537: 10.1016/j.brainres.2013.09.005 143-55 10.1016/j.brainres.2013.09.005 24041773

[14] : The role of retrieval mode and retrieval orientation in retrieval practice: insights from comparing recognition memory testing formats and restudying. *Cogn Affect Behav Neurosci*.16(6) : 10.3758/s13415-016-0446-z 977-990 10.3758/s13415-016-0446-z 27469235

[15] : Retrieving self-vocalized information: An event-related potential (ERP) study on the effect of retrieval orientation. *Brain Cogn*.92C: 10.1016/j.bandc.2014.10.011 123-132 10.1016/j.bandc.2014.10.011 25463147

[16] : Electrophysiological evidence for retrieval mode immediately after a task switch. *Neuroimage*.2015;108: 10.1016/j.neuroimage.2014.12.068 435-40 10.1016/j.neuroimage.2014.12.068 25562822PMC4334665

[17] : Preparation breeds success: Brain activity predicts remembering. *Cortex*.106: 10.1016/j.cortex.2018.04.009 1-11 10.1016/j.cortex.2018.04.009 29860188PMC6143439

[18] : Pre-retrieval event-related potentials predict source memory during task switching. *NeuroImage*.2019;194: 10.1016/j.neuroimage.2019.03.038 174-181 10.1016/j.neuroimage.2019.03.038 PMC654705330910727

[19] : Electrophysiological correlates of individual differences in strategic retrieval processing. *Cardiff University*.2010; Reference source

# Response

**Published:** 2019-10-09

**Authors:** Jane E. Herron

**Affiliations:** Cardiff University, UK

Thank you for your considered and interesting discussion of the cognitive processes and existing data regarding strategic retrieval. I have now noted and discussed the disconnect between pre-retrieval correlates of retrieval orientation and memory accuracy at the group level, and the discrepancy between this and item-related analyses which do link pre-retrieval ERPs with memory accuracy. I have also included your helpful observation that modulation of the target/nontarget retrieval ERP effects appear to have been driven by target rather than nontarget ERPs in the discussion. I have addressed each of the technical notes; automated artifact-detection thresholds have been added to the manuscript, the ALAS software tool and documentation have been uploaded to the accompanying Open Science Framework dataset (there is no published reference for this as far as I am aware), and the topographic maps and labelling have been amended as requested.

# Peer review: Reviewer response for version 1

**DOI:** 10.21956/wellcomeopenres.16759.r36336

**Published:** 2019-09-09

**Authors:** Rachael Elward

**Affiliations:** 1 London South Bank University, London, UK

The paper is the latest in a series of EEG experiments examining how cognitive control processes associated with memory retrieval are influenced by the prior completion of a Stroop Task. The Stroop Task is thought to require cognitive control resources which can become depleted from over-use. This depletion impairs the ability to engage in effective cognitive control strategies in subsequent tasks. Here, the authors employed a “task switching” paradigm which allows for an EEG investigation of the initiation of a retrieval orientation; a state that facilitates the retrieval of goal-relevant contextual details. The authors predict that the completion of a Stroop task will disrupt the engagement of a retrieval orientation because such a state requires cognitive control resources. This would be consistent with the idea that strategic retrieval processes are vulnerable to resource depletion. The results are consistent with this idea. These data have important implications for understanding the relationship between cognitive control processes and memory retrieval.  

 The study is well conducted with good power, sufficient trial numbers for all analyses and is technically sound. The manuscript is clearly written and with sufficient detail to allow for replication. 

 I have two issues that I would like to raise.

1. In Table 3, all the statistical results in the Target/Non-Target column are identical to that of the Target/New column. This looks to be an error? If so, this needs to be addressed before the article is indexed in it's final form. 
2. I would have liked to have seen a direct comparison of the magnitude of the EEG effects of interest between groups. When there is a significant interaction, such as in Group X Retrieval Task X Site interaction on page 7, all follow up tests involve repeated-measures ANOVAs within each group. From these follow-up tests, the authors report that there is evidence of a retrieval orientation in the control group, but no such evidence in the Stroop Group. A more direct comparison of the influence of the Stroop task on these EEG effects would be to conduct follow-up comparisons across the groups on subtraction waveforms (e.g. switch minus stay, or target minus non-target effects). Particularly, I would like to be able to make the statement “the difference in left parietal old/new effects for targets and non-targets was larger in the control group than in the Stroop group” but I am not sure that an analysis has been conducted that permits that statement. I can only state that there are significant effects of item type in the control group that are not significant in the Stroop group. If these outcomes are not reported because the group effects are not significant, then this impacts the interpretation of the results. The article is scientifically sound, however, without these analyses.

I confirm that I have read this submission and believe that I have an appropriate level of expertise to confirm that it is of an acceptable scientific standard, however I have significant reservations, as outlined above.

# Response

**Published:** 2019-10-09

**Authors:** Jane E. Herron

**Affiliations:** Cardiff University, UK

Thank you for drawing my attention to the statistical results presented in Table 3 which were duplicated in error. This table has now been amended, with the correct results entered for the Target/New contrast. This does not change the main finding, which is that the Target/New ERP effect was influenced by both the Stroop manipulation and switch/stay trial status. I have now also added follow-up comparisons of the ERP effects of interest across the groups on subtraction waveforms as requested. Significant effects of group were obtained in the analyses of i) pre-retrieval ERPs (Function-Draw) at mid-frontal sites between 700-1900ms, ii) target-new ERPs at the parietal maxima of this effect between 500-800ms, and ii) target-nontarget ERPs at the parietal maxima of this effect between 500-800ms.

# Peer review: Reviewer response for version 1

**DOI:** 10.21956/wellcomeopenres.16759.r36195

**Published:** 2019-08-30

**Authors:** Axel Mecklinger

**Affiliations:** 1 Experimental Neuropsychology Unit, Department of Psychology, Saarland University, Saarbrücken, Germany

The paper explores cognitive control process which occur prior to, during or after the recovery of memory episodes. It is assumed that retrieval orientations, brain states that bias the retrieval of specific memory contents over others play a central role during memory control. The study combines a memory task with either a Stroop task that makes demands on cognitive control resources or a control tasks that does not draw on cognitive control resources. Both tasks were conducted between the encoding and retrieval phase of the memory task. The critical manipulation was that in the retrieval phase two memory tasks were interwoven in a task-switching paradigm with pre-retrieval (task) cues specifying the memory task. The targeted memory contents changed after every two trials. This elegant design allows to explore electrophysiological (ERP) measures of the initiation of task-specific retrieval orientations upon presentation of the task cue on the one hand and of strategic retrieval, i.e. the prioritized retrieval of targeted over non-targeted memory contents otherwise. The question was how both processes depend on the availability of cognitive control resources (so-called cognitive control depletion).

 Remarkably, memory performance was not affected by the Stroop/control manipulation as one would expect under the assumption that the Stroop task but not the control task makes strong demands on cognitive control. However, strong effects were found for ERP measures of memory control. First, the control group showed strong and sustained ERP slow wave differences to the task cues on the first of the two memory trials (switch trials), which reflects the initiation of task specific retrieval orientations. Second, the control group also showed reliable ERP correlates of recollection for targeted memory contents over nontargeted contents, reflecting the successful prioritisation of targets over nontargets. Notably, both effects were virtually absent for participants who completed the Stroop task between the encoding and retrieval phase.    

 These results provide strong support for the author’s predictions, namely that performing the Stroop task before memory retrieval attenuates the ERP indices of the initiation of retrieval orientations, a proactive form of controlled memory retrieval and also diminished the ERP correlate of retrieving task relevant information, a recollection-related form of cognitive control.

 This is a well conducted and appropriately designed study that complements a series of three studies in which the author successfully applied a Stroop vs control group manipulation to explore how strategic retrieval processes and their ERP correlates are modulated by the availability of cognitive control reserves. Methods and results are clearly and accurately presented. The work is technically sound and the study design is highly appropriate to disclose the predicted electrophysiological effects. The implementation of a power analysis, the report of single trial numbers used for ERP averaging as well as the combined analyses of cued-locked and stimulus-locked ERPs are particularly strong points. Also, the behavioral and the subject ERP data are made available allowing the reproducibility of most of the results. For a complete reproduction of the ERP data the provision of the raw EEG data would have been desirable. Last but not least the discussion is very sound and well balanced and the main conclusions on the susceptibility of strategic memory retrieval to the availability of cognitive resources are always warranted by the data at hand. Laudably, alternative views (e.g. retrieval orientations are not necessarily a consequence of top town control process but can also arise from bottom up processes such as the ease with which nontarget information can be assessed, Rosburg & Mecklinger, 2017 ^1^) are adequately elaborated.

 One aspect that deserves further elaboration is the observation that effects of the Stroop/control manipulation were present for a variety of measures in the cue-locked and stimulus-locked ERPs but virtually absent for behavioral measures of memory accuracy. As acknowledged by the authors this disconnect between behavioral and ERP measures could imply that the effects of this manipulation were too subtle to be reflected in standard memory performance scores in particular when self-paced memory responses were employed as it was the case in the present study. Conversely, however, this disconnect also sheds light on an actual debate concerning the reliability of cognitive control (ego) depletion effects. Ego depletion effects have been criticized for being unreliable, not replicable or the sole result of inappropriate research practices such as p-hacking, publication bias or underpowered study designs. As revealed by a recent review paper (Friese et al. 2019 ^2^) there is in fact neither sufficient evidence for the presence or for the absence of ego depletion effects. While the absence of behavioral effects of the Stroop/control manipulation in the present study could be a direct reflection of insufficient power, the fact that robust ERP effects were obtained in the cue- and stimulus-locked analyses could suggest that these effects do exist but can only be disclosed with sufficiently sensitive measures, such as ERP measures of memory control processes. Further ERP studies with combined behavioral and ERP measures of cognitive control depletion are warranted to further explore this intriguing possibility.

I confirm that I have read this submission and believe that I have an appropriate level of expertise to confirm that it is of an acceptable scientific standard.

## References

[1] : Is Ego Depletion Real? An Analysis of Arguments. *Pers Soc Psychol Rev*.23(2) : 10.1177/1088868318762183 107-131 10.1177/1088868318762183 29591537

[2] : The costs of target prioritization and the external requirements for using a recall-to-reject strategy in memory exclusion tasks: a meta-analysis. *Psychon Bull Rev*.2017;24(6) : 10.3758/s13423-017-1256-1 1844-1855 10.3758/s13423-017-1256-1 28299598

# Response

**Published:** 2019-10-09

**Authors:** Jane E. Herron

**Affiliations:** Cardiff University, UK

Thank you for raising the interesting point regarding the ego depletion replication controversy. While this was not the primary motivation for the current study, I agree that the relative (and dissociable) sensitivities of the behavioural and the electrophysiological data in an appropriately powered study are interesting and could inform and influence this debate. A paragraph to this effect has been added to the discussion. The raw EEG data (*.bdf format) have been uploaded to the Open Science Framework and are now more clearly labelled (‘Raw EEG data’).
